# Inner egg shell membrane based bio-compatible capacitive and piezoelectric function dominant self-powered pressure sensor array for smart electronic applications†

**DOI:** 10.1039/d0ra02949a

**Published:** 2020-08-07

**Authors:** Qazi Muhammad Saqib, Muhammad Umair Khan, Jinho Bae

**Affiliations:** Department of Ocean System Engineering, Jeju National University 102 Jejudaehakro Jeju 63243 Republic of Korea baejh@jejunu.ac.kr

## Abstract

Flexible pressure sensors play a key role as an interface between the mechanical movements and electrical stimuli in smart skins, soft robotics, and health monitoring systems. However, conventional pressure sensors face several challenges in terms of their bio-compatibility, higher cost, and complicated fabrication process. This paper demonstrates a novel 5 × 5 bio-compatible capacitive and self-powered piezoelectric pressure sensor array using natural inner egg shell membrane (IESM). The proposed sensor array is supported by two different sensing modes, which are capacitive and piezoelectric function dominant self-powered mode. The capacitive mode can detect both static and dynamic pressures and the piezoelectric function dominant self-powered mode can be adopted to detect the dynamic pressure applied on the device. The fabricated device with a sensing area of 4 mm^2^ offered the sensitivity of 37.54 ± 1.488 MPa^−1^ in the capacitive pressure sensing range from 0 to 0.05 MPa. The device showed the response (*T*_res_) and recovery time (*T*_rec_) of 60 ms and 45 ms, respectively. The device achieves the sensitivity of 16.93 V MPa^−1^ from the sensing range of 0 to 0.098 MPa in the self-powered pressure sensing. These results depict that the proposed pressure sensor array will ensure a promising role in green, wearable, and soft electronic applications.

## Abstract 2

Inner egg shell membrane based bio-compatible capacitive and self-powered piezoelectric pressure sensor array is proposed, with capacitive sensitivity of 37.54 ± 1.488 MPa^−1^ (0 ≤ *P* ≤ 0.05 MPa) and piezoelectric pressure sensitivity of 16.93 V MPa^−1^ (0 ≤ *P* ≤ 0.098 MPa).

## Graphical abstract

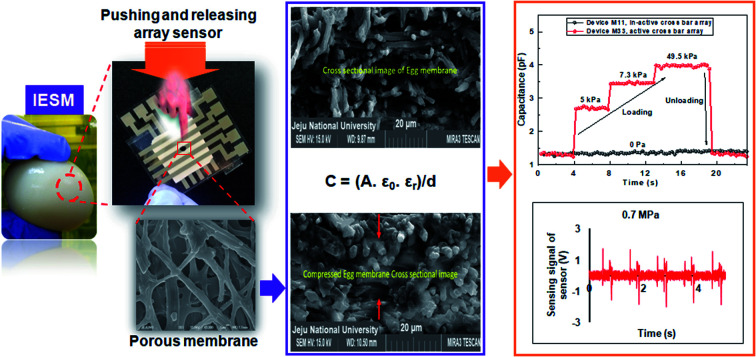

## 1. Introduction

Diversified soft and flexible pressure sensors have been extensively studied for a variety of potential applications, such as smart skins, automotive systems, biomonitoring devices, and soft robotics.^1–9^ Different pressure sensing technologies have been developed so far, based on the various operating mechanisms, such as piezoresistivity, piezoelectricity, and capacitance. All these mechanisms work on the principle of transforming mechanical motion into electrical stimuli.^10–17^ In the piezoresistive devices, flexible resistive sheets are employed as pressure-sensing layers integrated with a flexible circuitry.^18–20^ However, the metallic and metal oxide films used in piezoresistive pressure sensor face a possible failure because of cracks, and exhaustion due to the repetitive device operations. Similarly, the conventional self-powered triboelectric pressure sensors generally require an air gap for the contact separation in the vertical direction, which is a limitation for use in e-skins and other smart electronic applications.^21–23^ Hence, a self-powering pressure sensor is required without an air gap, so that it can be firmly attached with the monitoring body.

Among the various pressure sensing methods, a capacitive based sensing is a commonly used technique for pressure detection within a versatile sensing range.^24–27^ The sensor focuses on the idea of change in the capacitance of dielectric layer under external applied pressure. These sensors have the advantages in terms of signal repeatability, design simplicity, excellent stability, temperature insensitivity, and low power dissipation. However, the conventional capacitive based pressure sensors face few critical limitations in the practical applications. The capacitive based pressure sensors show small change in capacitance upon application of external pressure. Hence, the capacitive sensitivity becomes relatively low.^28,29^ In addition, the response (*T*_res_) and recovery time (*T*_rec_) of capacitive pressure sensor is also low. Large efforts have been made so far to overcome these limitations by modifying the dielectric layer in capacitive pressure sensors.^30^

Now a days, piezoelectric pressure sensors are getting more attention due to self-powered sensing and low fabrication cost.^31,32^ The piezoelectric pressure sensors work on the principle of generating dipole moments in the crystalline anisotropic materials under the applied pressure. These sensors have the shortcomings of incompatibility with soft and flexible substrates.^10,11,17^ Recently, the researches introduced new piezoelectric materials as flexible pressure sensing devices. However, the fabrication process is too complicated as compared with the other pressure sensing techniques.^33^ For these reasons, the previous pressure sensing techniques require the improvements in terms of their bio-compatibility, higher cost, and the complicated fabrication process.

After millions of years of evolution, the natural bio-materials have become equipped with perfect structures,^34,35^ which make them favorable to be used in smart electronic applications after artificial modifications.^36^ Therefore, the bio-compatible and environmental-friendly pressure sensing devices are urgently needed with the aim of achieving higher sensitivity, better stability, structure simplicity, flexibility, higher sensing range, and lower cost.^37,38^ However, a few bio-material based capacitive pressure sensors have been reported so far. Luo *et al.*, studied a capacitive micro-fabricated RF pressure sensor by employing air-filled parallel capacitor plates.^39^ The fabricated pressure sensor showed poor sensitivity in low pressure regime. Khalid *et al.*, employed a mixture of polylactic-*co*-glycolic acid (PLGA) and polycaprolactone (PCL), as a bio-degradable capacitive pressure sensing active layer suffering from challenge of poor stability and narrow pressure regime.^30^ Boutry *et al.*, reported a bio-compatible pressure sensor having high pressure sensitivity and intrinsic stretchability by utilizing the micro-structure of elastomer poly glycerol sebacate (PGS) as an active layer.^40^ Similarly, Kwon *et al.*, studied a microporous soft wearable pressure sensor by making use of elastomeric dielectric active layer.^41^ Although these sensing devices presented valuable output performances for the various environmental and biological pressures, however, they are facing some of the drawbacks as:^42^ (i) these sensors depend on the complicated cleaning room based fabrication tools. Moreover, the complex dielectric casting technique or micro-structuring leads to the complicated device structure.^30^ (ii) Battery is required to power the passive materials. Hence, a simple and uncomplicated fabrication process of the device is required along with improved sensing performance.

During the last few years, collagen fibril and amino acid based bio-materials attained a great attention because of their ease of availability and excellent sensitivity for different bio-compatible device applications. These fibrils and proteins are commonly available in human skin, the heart, and also in the inner egg shell membrane (IESM). IESM is the most abundant non-toxic and a low cost bio-material found between the egg shell and the egg white as illustrated in Fig. S1 in ESI.† The researchers have reported ESM as a cost-efficient, eco-friendly, and flexible printed electronic applications. Li Z. *et al.*, reported a highly efficient ESM based electrode material for supercapacitor applications.^43^ Similarly, Geng *et al.* presented the freestanding ESM based electrodes for supercapacitor applications along with oxygen evolution based reaction.^44^ The working phenomenon of the supercapacitor is different capacitive pressure sensor from many prospective.^45^ The capacitance in supercapacitors comes from the reversible charge accumulation upon the surface of electrode. Upon applying the potential difference, the cations and anions in the electrolyte move toward the surface of electrode materials (oppositely charged), hence a layer is formed near to separator. On the other hand, the capacitive pressure sensor works on totally different phenomenon. In the capacitive pressure sensor, the porous insulating layer in sandwiched between two electrodes. When an external pressure (instead of voltage) is applied across the sensing area, the thickness decreases. As results the capacitance across the electrode layer increases. The other factors may affect the capacitance of the capacitive pressure sensor to make the dipole creation. Khan *et al.*, used inner ESM for both the substrate and the humidity sensing active layer in a bio-compatible humidity sensor.^46^ The IESM carries various functions, like piezoelectric, triboelectric, capacitive, and humidity sensing, due to its nano-fibrous structure and proteins present in its structure.

The biocompatibility study of soluble egg shell membrane proteins (SEP) was made by Jia Jun *et al.*.^47^ In the reported work, they performed various analysis such as acute toxicity test, cytotoxicity test, cytotoxicity test, oral mucous membrane irritation test, and hemolysis test respectively to observe the biocompatibility of soluble egg shell membrane. The results showed that the SEP possesses excellent biocompatibility. Similarly, Feng Yi *et al.* conducted the amino acid and elemental analysis to study the biocompatibility of soluble egg shell membrane.^48^ The work showed that, the cystine losses during the amino acid analysis. As a result, the original structure of disulfide bonds changed to new disulfide bonds. The results in the cleavage of the originally structured disulfide bonds that are responsible for crosslinking. Hence, the IESM proved to be a potential biocompatible material for various green applications.

In this paper, an IESM based flexible, foldable, disposable, and bio-compatible 5 × 5 pressure sensor arrays with sensing areas of 0.25, 1, 2.25, and 4 mm^2^ are demonstrated, which are capable to work in two different sensing modes: capacitive and piezoelectric function based self-powered mode. Each sensor in an array from different rows and columns can be accessed without any cross talk. The IESM is composed of different types of fibrils and proteins. Highly porous nature of the IESM makes it an excellent candidate for capacitive pressure sensing application. Moreover, the hydrogen bonding between various micro-fibrils, proteins, and micro-fibrils and proteins leads to induce the polarization under external pressure. All these factors enhance the possibilities of both capacitive and piezoelectric properties in an IESM based pressure sensor.^49,50^ Among different sensing areas, 4 mm^2^ is selected because it has higher self-power generating capability. The selected IESM based capacitive pressure sensor offered the sensitivity of 37.54 ± 1.488 MPa^−1^ in the wide pressure range from 0 to 0.05 MPa. The sensor showed the response (*T*_res_) and recovery time (*T*_rec_) of 60 ms and 45 ms, respectively.

The proposed IESM carries both the piezoelectric and triboelectric dominant properties. Fig. S2 in the ESI† shows the photograph of the self-powered nano generator (NG) based on IESM (the detailed experimental descriptions using proposed NG and its mechanism are presented in Fig. S3 and S4 of the ESI,† respectively). Here, we use the piezoelectric dominant function of the IESM, which is more advantageous, as air gap is not useful for the various applications. Hence, the device can be firmly attached with the monitoring body for better sensitivity.^22^ Also, the humidity capturing will be minimized in the piezoelectric function dominant pressure sensors. The piezoelectric pressure sensor presented the comparable results with the previously fabricated bio-compatible sensing devices.^42,51–54^ The self-powered pressure sensor with the sensing area of 4 mm^2^ shows a fast response and recovery time (<10 ms), better sensitivity (16.93 V MPa^−1^) in a wide sensing range (0 ≤ *P* ≤ 0.098 MPa). However, these pressure sensors are suitable for dynamic pressure sensing applications, as the sensor shows zero pressure change when there is no tapping or stroke. On the other hand, the capacitive pressure sensor showed capacitive change against both static and dynamic pressures.

In conclusion, the IESM based capacitive pressure sensing devices can be adopted as necessary sensing device for static pressure sensing applications, while the IESM based self-powered pressure sensors can be selected for dynamic pressure sensing applications. For example, as a sensing scenario, after detecting a dynamic pressure using self-powered pressure sensor in sleeping mode without source, the IESM based sensor can detect static pressure using capacitance as a source. The work provides a comparison of fabricated capacitive pressure sensing device with previously reported bio-materials based capacitive pressure sensor. The fabricated device showed the higher response and recovery time as compared with other reported bio-compatible capacitive pressure sensors up to our knowledge. Furthermore, the proposed sensor has been applied for various real time applications such as wind and blow, and door vibration detection.

## 2. Materials and methods

### 2.1. Materials

Fresh hen eggs were purchased from the local market. Flexible PET substrate was purchased from Film Bank, Korea, having the thickness of 100 μm. Silver paste was purchased from Inktec Co. Ltd., Korea, and used without any further purification. Moreover, deionized water used for cleaning process, was purchased from Sigma Aldrich, Korea.

### 2.2. Preparation of IESM

**Fig. 1:**
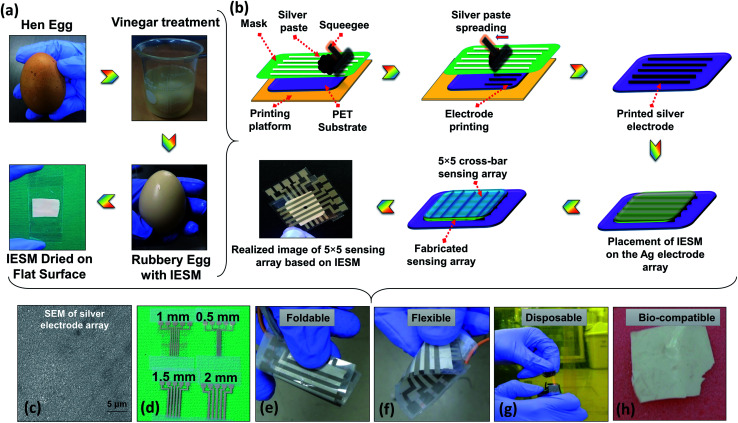
Illustration of IESM based pressure sensor. (a) Processing of IESM using white vinegar. (b) Typical screen-printing (screen printer: AMX-1240M) of the 5 × 5 electrode array. (c) SEM image of screen-printed silver electrodes. (d) Fabricated screen-printed silver electrode array on PET substrate having the width 0.5, 1, 1.5, and 2 mm respectively. The IESM based pressure sensor is: (e) foldable, (f) flexible, (g) disposable, and (h) bio-compatible.

As the IESM is tightly bonded with the outer egg shell, it is quite difficult to separate the IESM from outer egg shell directly. However, the outer egg shell can easily be extracted from the IESM by performing the vinegar treatment, as shown in Fig. 1a. The outer egg shell is composed of calcium carbonate (CaCO_3_). The vinegar contains nearly 4% of acetic acid. When we dissolved the hen egg in the vinegar, the reaction starts and the calcium carbonate egg shell dissolves. The carbon dioxide gas is released during the reaction of acetic acid with the calcium carbonate shell, and bubbles can be seen on the shell. The remained egg is entirely intact. We separated the egg shell membrane from the remained egg and washed carefully with DI water to avoid the effect of vinegar.

### 2.3. Fabrication

The fabrication process of the IESM based pressure sensor shown in Fig. 1b. The screen printer (AMX-1240M) was used to print 5 × 5 array electrodes using commercial silver paste as an ink on the PET substrate. After printing the array, the PET substrate was kept at room temperature for 5 min and then cured at 120 °C for 30 min. Fig. 1b shows the completely fabricated IESM based pressure sensor. The SEM image of silver electrode array in Fig. 1c ensures that, the electrodes are fabricated properly on the PET substrate. Fig. 1d shows the fabricated silver electrode array on the PET substrate. The width of the silver electrode array was kept 0.5, 1, 1.5, and 2 mm, respectively. The partially wet IESM based capacitive and self-powered pressure sensing layer was carefully placed on the bottom PET substrate. Finally, 5 × 5 top electrode array was placed on the IESM in cross bar pattern. The sandwiched IESM was dried at room temperature for 24 hours to remove the air bubbles. The fabricated pressure sensor array was entirely encapsulated to avoid the humidity effect present in the environment. Fig. S1† insures the presence of IESM between egg albumen and outer egg shell membrane. The IESM consists of well-ordered collagenized micro fibrils and proteins. Fig. 1e–h show the foldable, flexible, disposable, and bio-compatible properties of the IESM based pressure sensor. These properties ensure that the IESM based soft sensing device can be firmly attached with any monitoring body to measure the applied pressure along with strong bio-compatibility usually detrimental for the environmental-safety. The fabricated pressure sensor can be easily disposed of by incineration process within few seconds without any contamination.

### 2.4. Pressure sensing analysis

To test the static response of IESM based capacitive pressure sensor, a pressure sensing setup containing homemade highly calibrated pressure measuring unit, RA9 commercial pressure sensor (sensing range: 5 gf to 4000 gf, mechanical tolerance ≤ 50 μm, durability 2 000 000 strokes for 100 g, response time < 10 μs, operating temperature 20 °C to 60 °C, and output tolerance Max. 20%), the NF ZM2372 LCR meter for capacitance measurements, and calibrated weights were used (in ESI,† the detailed description of RA9 application setup is shown in Fig. S5† and the capacitance characterizing setup is illustrated in Fig. S6†). The reference sensor was connected with an Arduino nano (for controlling) and a PC (for sensing data acquisition). Fig. S7† shows the calibration test of RA9 commercial pressure sensor used as a reference sensor. It is shown that, the reference sensor was matched with the pressure exerted by calibrated weights. To test the dynamic response of self-powered piezoelectric pressure sensor, a linear servo motor (IRROBOT, L12-30PT-4), RA9 commercial pressure sensor, and Agilent DSO7052B oscilloscope (input impedance of 1 MΩ) were used as shown in Fig. S8a,† and its conceptual structure is illustrated in Fig. S8b.† ZM237x application software was used for data logging of capacitance measurements. The reference sensor showed the voltage in digital form from 0 to 1023 units as the external pressure was increased. The digital values were converted to analog voltage by the digital to analog conversion (DAC) phenomenon, and the final pressure was attained.

### 2.5. Characterizations

**Fig. 2:**
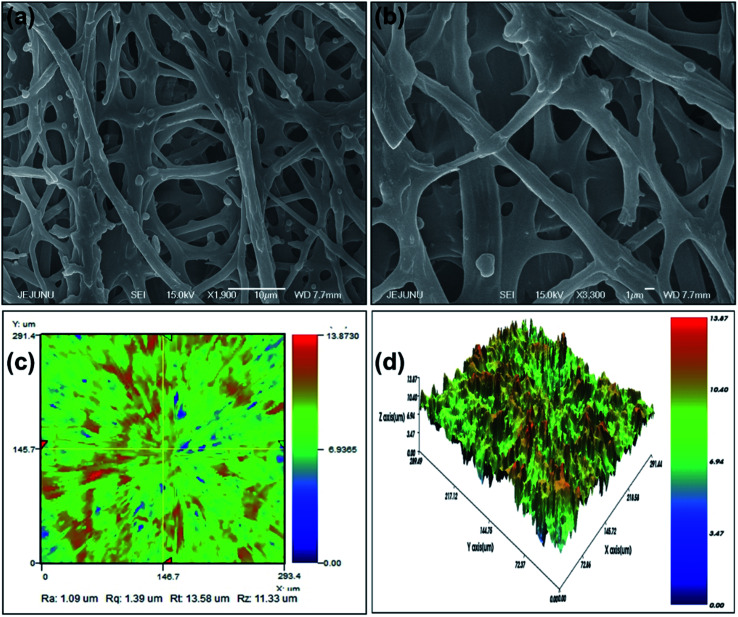
Analysis of the IESM used in the capacitive and self-powered piezoelectric pressure sensor. (a) FESEM image of IESM at 10 μm scaling. (b) FESEM image of IESM at 1 μm scaling. (c) 2D nano profile of IESM. (d) 3D nano profile of IESM.

**Fig. 3:**
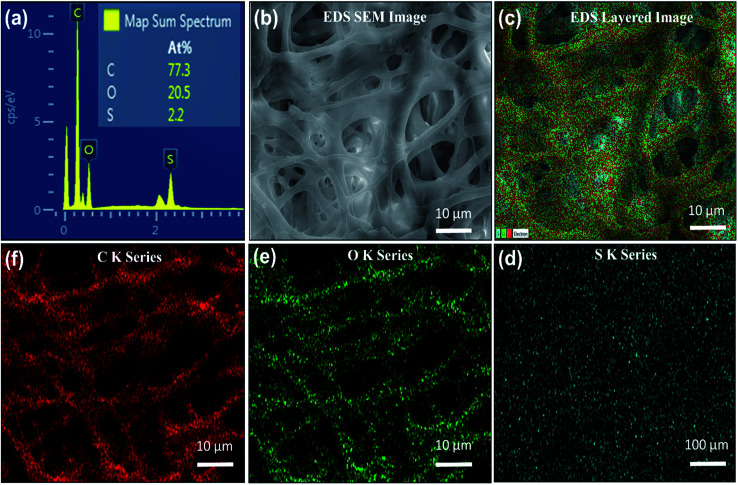
EDS mapping analysis of the IESM used in the capacitive and self-powered piezoelectric pressure sensor. (a) EDS representation of the IESM, the inset image shows the elemental composition percentage in the IESM. (b) EDS mapping based SEM image of IESM. (c) EDS based layered image, the elemental mapping of the IESM showing, (d) S K series, (e) O K series, and (f) C K series.

To study the chemical and structural composition of IESM as mentioned in Fig. 2 and 3, various characterization techniques are applied. The detailed morphology analysis of IESM was performed using scanning electron microscopy (SEM JEOL JSM-7600F) by coating the membrane with platinum sputter. The NZ-2000 Universal non-contact surface profiler was used to analyze the 2D and 3D nano-profile of the IESM to study the surface roughness and film thickness in the phase shifting interferometry mode (PSI). TESCAN MIRA 3 STEM was used to perform the EDS mapping of IESM to investigate the detailed elemental composition. The functional groups of the IEMS was analyzed by Fourier transform infrared spectroscopy (FRIR, Thermo fisher Scientific Nicolet 6700 spectrometer). Robust mini linear servo actuator (Model L12-30PT-4 and L12-20F-3) was used to apply the constant pressure on the capacitive and piezoelectric pressure sensor array.

## 3. Results and discussions

### 3.1. SEM images

Fig. 2a and b depict the highly porous network of IESM with the magnification of ×500 at the scaling of 1 and 10 μm, respectively. It is clearly shown that the natural IESM consists of small pores and cavities with the diameter ranging from 0.5 to 1.5 μm, respectively.

### 3.2. 2D and 3D nano-profile

Fig. 2c and d present the 2D and 3D nano-profile of IESM respectively. Fig. 2c shows the surface roughness of IESM to be 1.35 μm, while the thickness varies between 18 to 21 μm. Fig. 2d shows that, the IESM has highly porous multilayer crosslinked structure having large number of micro-fibrils and proteins. Because of highly porous structure of IESM, an excellent change in capacitance was observed due to the large displacement in the thickness when an external pressure is applied.

### 3.3. EDS mapping

The EDS energy dispersive analysis ensures the existence of carboxyl and carbonyl groups, and amino acids in the IESM that are required to make the highly collagenized micro-fibrils and proteins in the membrane. The EDS analysis of the X-ray spot profile clearly illustrates the S, O, and C peaks in IESM, as shown in Fig. 3a. Fig. 3b expresses the EDS based SEM image with the scaling of 10 μm. The EDS layered image of IESM is shown in Fig. 3c. The layered image ensures that the IESM contains sulfur series having atomic percentage 2.2%, oxygen series 20.5%, and carbon series 77.3% as shown in Fig. 3d–f, respectively.

### 3.4. FTIR spectra analysis

**Fig. 4:**
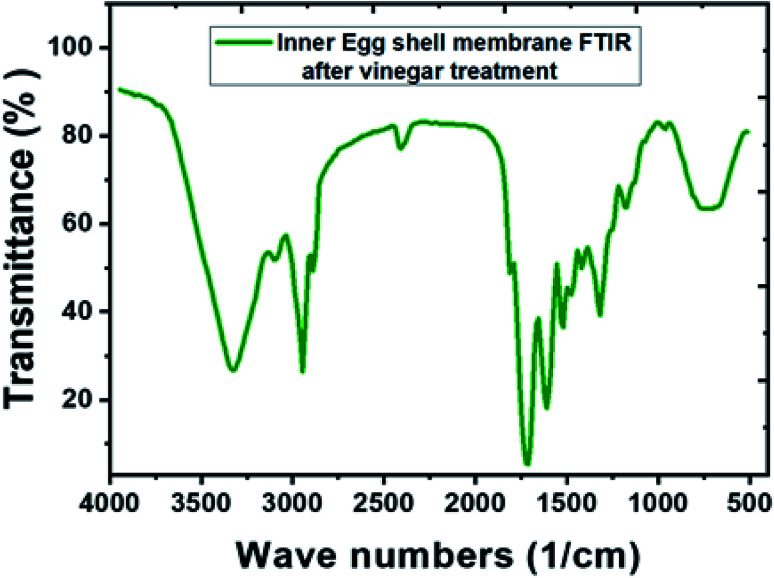
FTIR spectra of IESM after the vinegar treatment to remove the egg outer shell.

After the vinegar treatment, functional groups of the IESM were analyzed by the Fourier transform infrared spectroscopy (FTIR) as shown in Fig. 4. On the other hand, the FTIR analysis of the untreated IESM was reported by Hsieh *et al.*, which shows that the peak of the IESM elaborated as:^55^ 661 cm^−1^ represents C–S band, 1109 cm^−1^ represents amine C–N band, 1451 cm^−1^ represents CH_2_ band, 1536 cm^−1^ represent amide N–H band, 1643 cm^−1^ represents amide C=O band, 2929 cm^−1^ represents C–H band, and 3274 cm^−1^ represents O–H and N–H band. As compare to Fig. 4, it can be seen that there is no significant change in the peaks of IESM after vinegar treatment. All these features conforms the purity of the IESM after the vinegar treatment.

### 3.5. Capacitance and self-powered pressure sensing

**Fig. 5:**
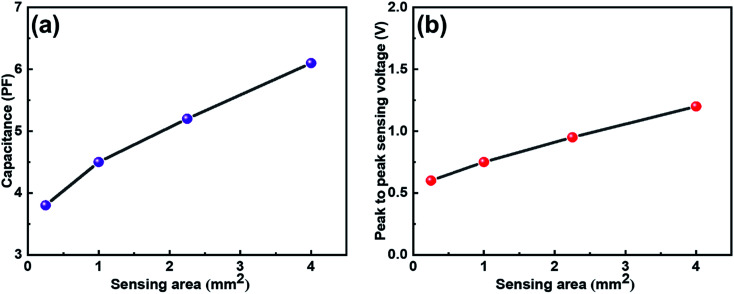
(a) Capacitive change against the various sensing areas. It is shown that, the capacitance increases with increasing sensing area. (b) Peak to peak sensing voltage against various sensing areas. It is shown that, the voltage increases with increasing sensing area.

The capacitance *vs.* sensing area graph of IESM based capacitive sensor array is shown in Fig. 5a. It is shown that, the capacitance of capacitive pressure sensor increased linearly with increasing sensing area. Fig. 5b shows that, the peak to peak sensing voltage showed slightly increasing behavior when the sensing area was increased from 0.25 to 4 mm^2^, respectively. Fig. S9† shows that, the sensing signal decreased from 1.2 to 0.5 V, when the sensing area was decreased from 4 to 0.25 mm^2^, respectively. It can be seen that, the larger area can get higher self-powering signal than the small one. For this reason, 4 mm^2^ sensing area was selected for IESM based capacitive and self-powered piezoelectric dominant pressure sensor.

### 3.6. Capacitive pressure sensor array

**Fig. 6:**
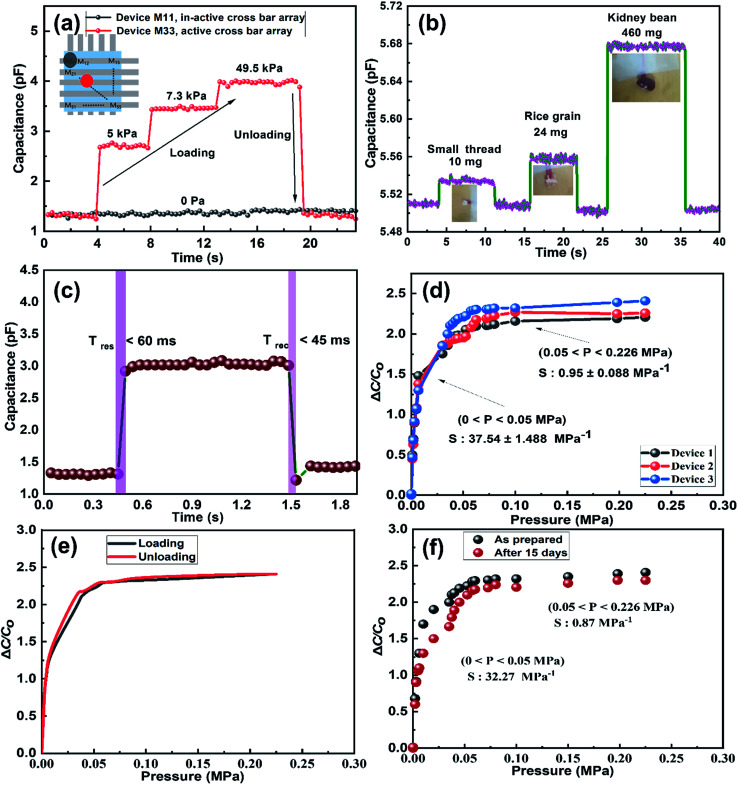
The static response of IESM based capacitive pressure sensor. (a) Shows the capacitive response against three different pressures; 5 kPa, 7.3 kPa, and 49.5 kPa exerted on IESM based capacitive pressure sensor active cross bar array (device M33), and non-active cross bar array (device M13). The inset figure shows the schematic illustration of 5 × 5 IESM based pressure sensor. (b) Capacitive response of IESM based pressure sensor against loading and un-loading of tiny piece of thread (∼10 mg), grain of rice (∼24 mg), and a kidney bean (∼460 mg). (c) Response and recovery time of capacitive pressure sensor. (d) Shows the relative capacitance change *vs.* pressure graph for sensitivity measurements. (e) Capacitive response of IESM based capacitive pressure sensor for loading and unloading cycles. (f) Shows the effect on the device efficiency after 15 days of the incubation. The results depict that, the device showed the negligible sensitivity change.

This section provides the experimental results about the static response of the 5 × 5 IESM based capacitive pressure sensor array with the 4 mm^2^ sensing area. The idea of the present work is to determine the resultant capacitance change against an external applied pressure. Fig. 6 depicts the sensing performance of IESM based capacitive pressure sensor. The sensitivity (*S*) of the capacitive pressure sensor is defined by eqn (1) (ref. 26) as1
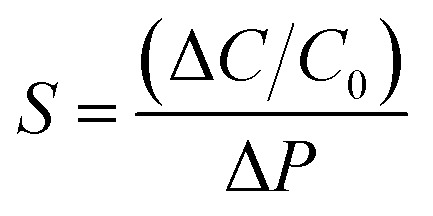
Here, Δ*C* represents the capacitance change against the applied pressure, *C*_0_ represents the baseline capacitance, and Δ*P* is the change in applied pressure on the device. Fig. 6a shows the real time values of the fabricated pressure sensing device by loading/unloading various pressures of 5, 7.3, and 49.5 kPa, respectively. In Fig. 6a, *M*_11_ and *M*_33_ represent the in-active (non-pushing) and active (pushing) sensor elements in the crossbar array, respectively. The inset image in the Fig. 6a shows the structural illustration of cross bar array. These responses demonstrate that the IESM based capacitive pressure sensor show stable sensing behavior with better sensitivity and significant reproducibility. Similarly, the real time responses against extremely low pressures applied by a tiny piece of thread (10 g), rice grain (24 g), and kidney bean (460 g) proves the excellent sensitivity of an IESM pressure sensor in the lower pressure region as shown in Fig. 6b. The response (*T*_res_) and recovery time (*T*_rec_) of the capacitance of the proposed sensor are found to be 60 ms and 45 ms, respectively as shown in Fig. 6c. As compared with the literature, the measured *T*_res_ and *T*_rec_ is higher than the previously reported bio-compatible capacitive pressure sensing devices as shown in Table 1.^30,39–42^ The faster response and recovery time can be expressed by the viscoelastic property of the IESM. Since the IESM possess the least elastic resistance in the low pressure region, it shows excellent response and recovery time. However, the response and recovery time may show a significant decrease in the high pressure regime due to the increased elastic resistance of IESM.^5,56^

**Table 1:** The comparison of the fabricated IESM based capacitive pressure sensor with the previously reported bio-compatible capacitive pressure sensors

No.	Bio-material	Sensitivity	Sensitivity range	*T*_res_	*T*_rec_	Ref.
1.	PGS	0.76 ± 0.14 kPa^−1^	*P* < 2 kPa	—	—	40
2.	PLGA-PCL	0.863 ± 0.025 kPa^−1^	0 < *P* ≤ 1.86 kPa	251 ms	170 ms	30
**3.**	**IESM**	**37.54 ± 1.488 MPa** ^**−1**^	**0 < *P* ≤ 0.05 MPa**	**60 ms**	**45 ms**	**This work**

**Fig. 7:**
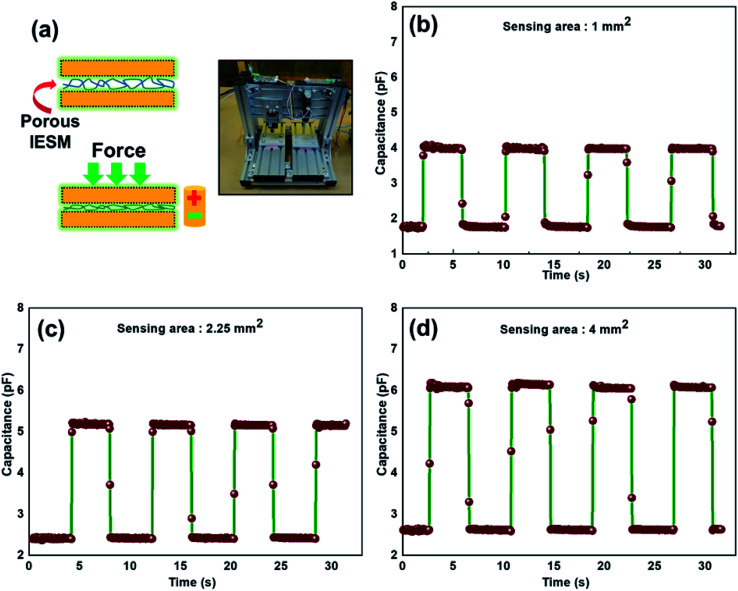
(a) Schematics of the IESM based capacitive pressure sensor with and without applied pressure. The inset shows the photograph of linear servo motor. Capacitive change against sensing areas of (b) 1 mm^2^, (c) 2.25 mm^2^, and (d) 4 mm^2^, respectively.

Fig. 6d presents the sensitivity of the three sensing devices. It can be seen that the two linear regions exist under the application of external pressure on an active sensor array. The IESM based capacitive pressure showed the sensitivity of 37.54 ± 1.488 MPa^−1^ in the wide pressure range from 0 to 0.05 MPa. When the external pressure was increased, the sensitivity of fabricated pressure sensor decreased to 0.95 ± 0.008 MPa^−1^. However, it is comparable with the previously reported biocompatible capacitive pressure sensing devices.^39–42^ The decreased sensitivity in a high pressure region is most probably due to breaking hydrogen bonds between collagenized fibrils and proteins at high external pressure.^5,56^ In ESI, Fig. S10† shows the sensitivity of IESM based capacitive pressure sensor against the applied force (N). The sensitivity of IESM based capacitive pressure sensor was 10.2 N^−1^ in the range from 0 to 0.2 N. The restorability test of IESM based capacitive pressure sensor is shown in Fig. 6e. It is shown that, the unloading of the capacitive sensor is well matched with the loading process of the sensor. The stability test of the fabricated capacitive pressure sensor was performed after 15 days as shown in Fig. 6f. The sensor showed the sensitivity of 32.27 MPa^−1^ in the wide pressure range from 0 to 0.05 MPa. Similarly, the sensor showed the sensitivity of 0.87 MPa^−1^ in the high pressure range 0.05 ≤ *P* ≤ 0.226 MPa. Table 1 shows the comparison between the fabricated IESM based pressure sensor array with the previously reported bio-material based capacitive pressure sensors. It shows that the fabricated IESM based capacitive pressure sensor presents better sensitivity, fast response and recovery time, and wide detection range (see movie file, ESI movie 3.mp4† to get IESM based capacitive pressure sensing). Fig. 7a shows the schematic of the IESM based capacitive pressure sensor with and without applied pressure. A constant pressure was applied with the robust mini linear servo actuator shown in Fig. 7a. The capacitive change against the sensing areas of 1, 2.25, and 4 mm^2^ is shown in Fig. 7b–d. It can be seen that the capacitance increases by increasing the sensing area. The capacitance with the sensing area of 1 mm^2^ is 4.2 pF. When the sensing area was increased to 2.25 and 4 mm^2^, the capacitance increased to 5.2 and 6.2 pF, respectively.

### 3.7. Piezoelectric function dominant self-powered pressure sensor array

**Fig. 8:**
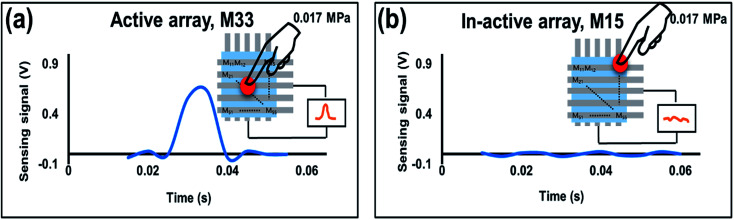
(a) Dynamic voltage response for active sensing array (*M*_33_) against the external pressure of 0.0175 MPa. (b) Device showed negligible response, when the pressure is applied across in-active sensing array (*M*_15_).

The paper was also evaluated the dynamic response of 5 × 5 IESM based self-powered piezoelectric function dominant pressure sensor array with the 4 mm^2^ sensing area. The idea of the present work is to determine the resultant voltage signal when a dynamic pressure is applied. The voltage response of the fabricated pressure sensor against various pressures is shown in Fig. S11 in ESI.† The pressure sensor showed an increment from 0.78 V to 2.05 V by varying the pressure from 0.02 MPa to 0.7 MPa as shown in Fig. S11a–e.† Hence, the performance of the proposed sensor showed an increase against the increasing magnitude of applied external pressure. Fig. S11f† presents the sensing voltage plotted against the pressure applied. The sensitivity of the fabricated sensor in the pressure region from 0 to 0.098 MPa was measured to be 16.93 V MPa^−1^. The sensitivity of the voltage signal against applied pressure decreased to 0.098 V MPa^−1^ in a high pressure region (0.098 ≤ *P* ≤ 0.7 MPa). In ESI, Fig. S12† shows the sensitivity graph of piezoelectric function dominant pressure senor against force in newton. The piezoelectric function dominant pressure sensor offered the sensitivity of 2.7 V N^−1^ in the sensing rage of 0 to 0.6 N, while it has achieved the sensitivity of 0.10 V N^−1^ when the sensing range lies from 0.6 to 2.56 N. Fig. 8 presents the real-time dynamic response of the fabricated 5 × 5 piezoelectric sensor array. It is demonstrated that the self-powered sensor array showed a sensing signal of 0.625 V when an external pressure of 0.017 MPa is applied across the active sensing array (*M*_33_) as shown in Fig. 8a. Fig. 8b shows the negligible change in the sensing signal, when pressure is applied across in-active sensing array (*M*_15_). Fig. S13a† shows the IESM based sensor matrix having active and non-active arrays. When an external pressure of 0.01 MPa is applied across the active sensing array, the sensor shows the sensing signal of 0.75 V as shown in Fig. S13b in ESI.† All non-active sensing arrays showed negligible variation in voltage signal against external applied pressure as shown in Fig. S13c–f.† Fig. S13g–k† present the active and in-active sensing arrays against 0.03 MPa external pressure. The response and recovery time of the proposed sensor was measured to be <10 ms that make the sensor an efficient device for dynamic pressure sensing measurements (see Fig. S14 in ESI† and movie file, ESI movie 4.mp4† to get IESM based piezoelectric function dominant self-powered sensing).

### 3.8. Mechanism of the proposed sensor array

**Fig. 9:**
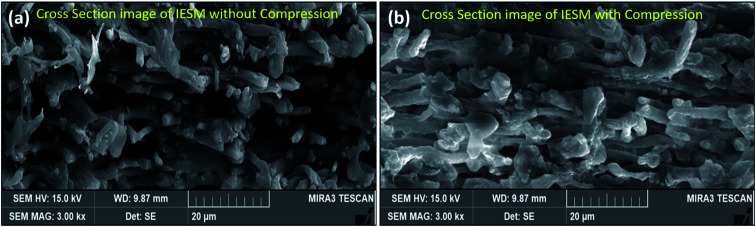
Cross-sectional SEM image of IESM in (a) normal and (b) compressed state.

For the mechanism of the capacitance changes in the proposed sensor upon the application of external pressure, the capacitance of the proposed sensor can be calculated by eqn (2) (ref. 30) as2
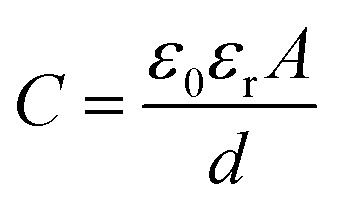
where, *A* is a sensing area of the IESM in meter square, *ε*_r_ represents the dielectric constant, *ε*_0_ is the dielectric constant of vacuum (*ε*_0_ = 8.54 × 10^−12^ F m^−1^), and *d* is the thickness of the IESM. As there is an inverse relation between the capacitance *C* and the thickness *d*, the capacitance changes upon the application of external pressure due to the change in the thickness *d* of the IESM. The change in the thickness of the IESM is predominant due to the porous nature of the micro-fibrils and proteins present in the IESM.^57,58^ The cross-sectional SEM images in Fig. 9a and b show the comprehensive picture of the porous structure of the IESM. Fig. 9a depicts the cross-sectional SEM image of IESM in normal state, showing pores in its structure. When the external pressure is applied, the pores come closer to each other, leading to the significant decrease in the thickness *d* of membrane, and result in an increase in the capacitance of the capacitive sensor as shown in Fig. 9b. There are many factors which may cause an increase in the capacitance of the IESM, when an external pressure is applied. The IESM is composed of the various collagen fibrils (collagen I, V, and X), and proteins (sialoprotein, osteoprotein, and keratin).

**Fig. 10:**
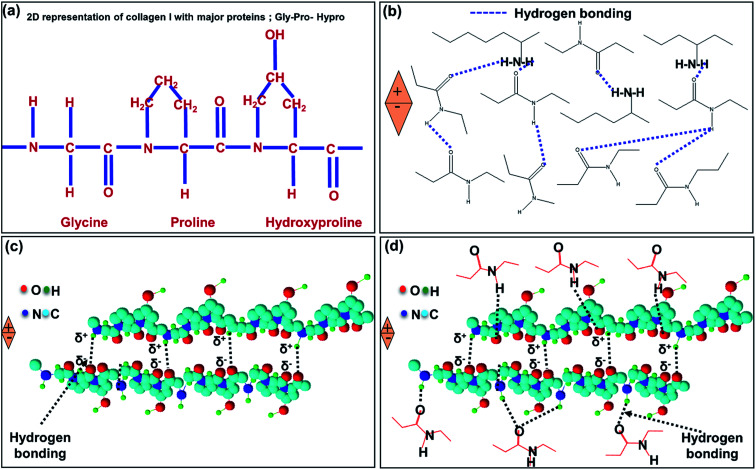
(a) 2D representation of collagen I with major proteins; GLY-PRO-HYP motif. (b) Hydrogen bonding interaction between collagenized micro-fibrils. (c) Hydrogen bonding interactions between different amino acids. (d) Hydrogen bonding between collagen fibrils and proteins.

Fig. 10a presents the primary structure of type I collagen showing the amino acid motif (Gly-X-Y), where X represents the proline (pro) and Y represents hydroxyproline (HyPro) respectively.^58–60^Fig. 10b ensures that the presence of hydrogen bonding between –CONH– in the collagen fibrils of the IESM lattice structure. When an external pressure is applied across the IESM, there is a possibility to develop the poles across the collagenized micro-fibrils that lead to an increase in the capacitance of the membrane. Fig. 10c shows the presence of hydrogen bonding and/or van der Waals interactions between the proteins present in the IESM. Due to these interactions, the amino acids present in the lattice structure are aligned in same direction. Hence, there is a possibility of poles creation under the influence of external pressure. Also, there is a possibility of hydrogen bonding between highly collagenized micro-fibrils and amino acids present in the IESM as shown in Fig. 10d. The polarization occurs because of unidirectional alignment of micro fibrils and proteins under the influence of external pressure. All these factors may lead to an increase in the capacitance of the IESM when an external pressure is applied.^50,61^ Similarly, the mechanism for electrical nano-generation in a porous IESM based self-powered piezoelectric pressure sensor can be explained by the various hydrogen bonding interactions between micro-fibrils and proteins present in IESM.^50,61^ Dipole orientation of collagens and proteins as discussed in the above section leads to the piezoelectric nanogeneration in the IESM lattice structure. The polarization in the lattice structure creates a sufficient piezoelectric potential difference between top and bottom electrodes under the influence of external pressure that drives the electric current in an external circuit.^61–64^ Although, the voltage sensing signal in triboelectric function dominant devices is higher, however, the sensitivity and encapsulation are more dominant than the higher voltage with air gap. Hence, this paper utilized the piezoelectric function dominant mode without air gap.

## 4. Applications

The high sensitivity and fast response and recovery time in the IESM based capacitive pressure sensor make it suitable for low static pressure measurements. The proposed IESM based pressure sensor is employed for smart electronic applications. Previously, although the researchers reported bio-compatible pressure sensors for wearable and soft applications. However, it is an immense need to present the bio-compatible and environmentally friendly sensing devices for daily used pollution free applications. In the present work, the wind, blow, and door applications were reported to ensure the extraordinary sensitivity of the IESM towards small external force.

The wind and blow detection applications utilizes a commercially available high precision digital pressure sensor (Sensirion, EK-P4) used as reference sensor to measure pressure (in pascal). The reference sensor EK-P4 was typically used for wind and blowing monitoring so that, the IESM based capacitive pressure sensing performance can be judged in low pressure regime. On the other hand, the piezoelectric function dominant pressure sensors are more advantageous for dynamic pressure change, due to their self-powering ability and quick response. This work presented the self-powered piezoelectric function dominant pressure sensor for vibration detection produced from closing the door.

### 4.1. Wind blowing detection

**Fig. 11:**
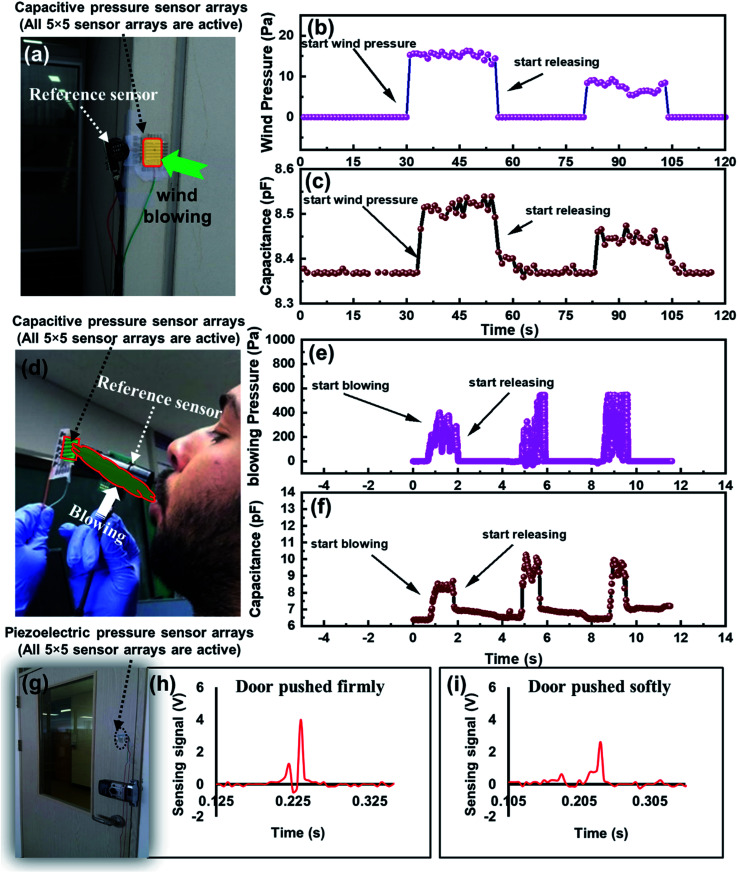
(a) Photograph for wind detection using IESM based capacitive pressure sensor. (b) Wind pressure measurement using the digital pressure sensor (EK-P4, Sensirion), used as a reference sensor. (c) Real time capacitive response of IESM based capacitive pressure sensor against the wind blowing in the atmosphere. (d) Photograph for blowing detection from the mouth. (e) Real-time pressure measurement exerted by the blowing from mouth using reference sensor. (f) Capacitive-response of IESM based capacitive pressure sensor against the blowing from the mouth. (g) Photograph door detection using IESM based piezoelectric function dominant pressure sensor. (h) Voltage sensing signal of self-powered pressure sensor when the door pushed firmly. (i) Voltage sensing signal, when the door closed in soft manner.

To demonstrate the real time performance of an IESM based capacitive and self-powered piezoelectric function dominant pressure sensor array, the device was implemented to monitor the wind pressure measurements as shown in Fig. 11. All 5 × 5 sensors arrays were made active for obtaining better sensitivity. EK-P4 digital pressure sensor, used as a reference sensor to measure the wind pressure is shown in Fig. 11a. Fig. 11b shows the real-time wind pressure measurement using reference pressure sensor. The wind pressure against various wind pressures was accurately monitored, using the fabricated capacitive pressure sensor as shown in Fig. 11c. It can be seen that, when the wind pressure was near 15.2 Pa, the capacitive pressure sensor showed the maximum capacitance of 8.54 pF. When the wind pressure dropped to 9.3 Pa, the capacitance decreased to 8.46 pF. The capacitance change was due to the increased external pressure exerted by strong and slow winds, respectively.

### 4.2. Air blowing detection from the mouth

The fabricated capacitive pressure sensor was successfully implemented to detect the air blowing from the mouth. Fig. 11d shows the photograph for wind blowing detection from the mouth. EK-P4 digital pressure sensor was used as a reference sensor to measure the blowing pressure from the mouth as shown in Fig. 11e. Fig. 11f depicts the capacitive change of fabricated pressure sensor against the changing blow pressure. It can be seen, when the blowing pressure varied from 390 to 547 Pa, the capacitance of our fabricated capacitive pressure sensor varied from 8.48 to 10.7 pF. The results show that the fabricated capacitive pressure sensor has a capability to detect a series of pressures, and exert by different wind blow conditions due to its high sensitivity in the low pressure regime.

### 4.3. Door moving detection

To demonstrate the monitoring capability of IESM based self-powered piezoelectric pressure sensor for door detection, the sensor was fixed at the door as shown in Fig. 11g. Fig. 11h and i show the output sensing signals, when the door was closed in the fast or the slow manner. The sensing signal generated by the pressure sensor was due to the vibrations produced in the door. When the door was closed firmly, the voltage sensing signal was higher because of the higher vibrations generated in the door, and *vice versa*. The results show that the IESM based self-powered pressure sensor has an excellent capability of door detection against the dynamic pressure due to the high sensitivity in the low pressure regime. We are confident that the fabricated IESM based pressure sensing device will find the intense applications in the field of e-skins, soft robotics, and healthcare technology. The work thus, opens the gateway for economical, bio-compatible, and all printed organic pressure sensors.

## 5. Conclusion

In the paper, we successfully demonstrated a novel bio-compatible IESM based 5 × 5 pressure sensor array. We examined the device performance with various the sensing areas of 0.25, 1, 2.25, and 4 mm^2^ respectively. Finally, the sensing area of 4 mm^2^ was selected because of high capacitance and sensing signal values as compared with the other ones. The selected pressure sensing device was capable of working in two different sensing modes: capacitive mode and self-powered piezoelectric function dominant mode. In capacitive sensing mode, the device showed the sensitivity of 37.54 ± 1.488 MPa^−1^ in the wide pressure region (0 ≤ *P* ≤ 0.05 MPa). The proposed device showed the response (*T*_res_) and recovery time (*T*_rec_) of 60 ms and 45 ms, respectively, which was faster than the reported bio-compatible capacitive pressure sensors. For dynamic pressures, IESM based piezoelectric function dominant pressures sensors were more promising because of their self-powering property, along with the fast response (*T*_res_) and recovery time (*T*_rec_). The fabricated self-powered piezoelectric pressure sensor array showed the sensitivity of 16.93 V MPa^−1^ in the wide pressure region (0 ≤ *P* ≤ 0.098 MPa), which is comparable with the previously reported bio-compatible pressure sensors. Moreover, the device does not require an air gap for contact separation, thus can be firmly attached with the monitoring body. This approach offers a roadmap toward bio-compatible, environmental-friendly, and self-powered potential electronic applications.

## Conflicts of interest

The authors declare no conflict of interest.

## Supplementary Material

RA-010-D0RA02949A-s001

RA-010-D0RA02949A-s002

RA-010-D0RA02949A-s003

RA-010-D0RA02949A-s004

RA-010-D0RA02949A-s005
