# Supplementary material for: Inner egg shell membrane based bio-compatible capacitive and piezoelectric function dominant self-powered pressure sensor array for smart electronic applications
Source: RSC Adv. 2020 Aug 7;10(49):29214–27. doi: 10.1039/d0ra02949a (PMC9055931; doi:10.1039/d0ra02949a)
Supplement: RA-010-D0RA02949A-s001 [file RA-010-D0RA02949A-s001.pdf]

Electronic Supplementary Material (ESI) for RSC Advances.
This journal is © The Royal Society of Chemistry 2020

Supplementary Information Inner egg shell membrane based bio-compatible capacitive and piezoelectric function dominant self-powered pressure sensor array for smart electronic applications

Qazi Muhammad Saqib, Muhammad Umair Khan, and Jinho Bae

1. Structure of hen egg

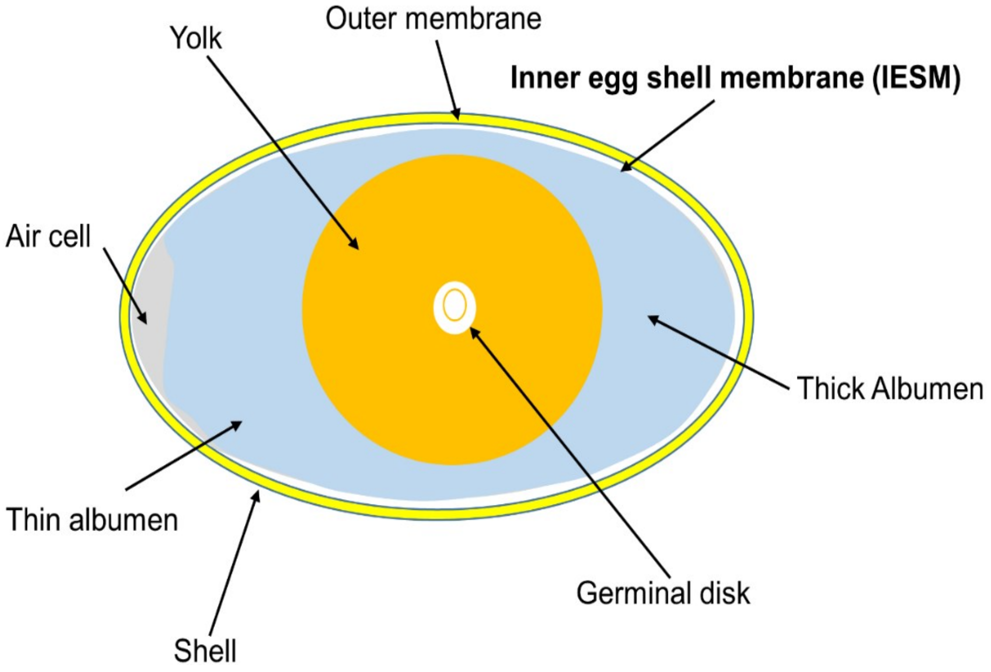

Fig. S1. Structure of hen egg. IESM between egg white and egg shell consists of well-ordered collagenized micro fibrils and proteins.

2. Piezoelectricity and triboelectricity of IESM

The combined effect of triboelectricity and piezoelectricity can be demonstrated for mechanical to electrical conversion in the IESM. For the device fabrication process, the IESM was cut into 2×2 cm^2 and then covered by copper tape on the both sides. The copper tapes acts as top and

bottom electrodes. The device was covered with the polypropylene tape to avoid from environmental effects. The photograph of the fabricated device is shown in Fig. S2. A pneumatic air cylinder (SHINYEONG MECHATRONICS CO., LTD, C32B 16-50, Max Press 0.7 MPa) was used as a stepping motor with the stepping of 10 steps/sec. Fig. S3 shows five type of structures to study both piezoelectricity and triboelectricity effects in the IESM based sensing devices. Fig S3a presents the IESM based piezoelectric function dominant device. Fig. S3b shows the triboelectric function dominant device. It can be seen that, the device showed higher voltage as comparted with the piezoelectric function dominant device. In pressure sensing device, we employed piezoelectric function dominant phenomenon to vanish the air gap for better capacitive and piezoelectric sensitivity along with minimizing the humidity capturing ability for better life time. Fig. S3c showed the hybrid structure of IESM nanogeneration. The results clearly differentiated the contribution of piezoelectricity and triboelectricity in triboelectric function dominant devices. The voltage signals were enhanced when hybrid structure of two IESM based piezoelectric devices were attached back to back as shown in Fig. S3d. Fig. S3e showed that, very low signals are generated when air gap is induced between the sensing layers [1]. (See movie files, Supplementary movie 1 and 2.mp4 to get piezoelectric function dominant and triboelectric function dominant devices, using stroke test setup.)

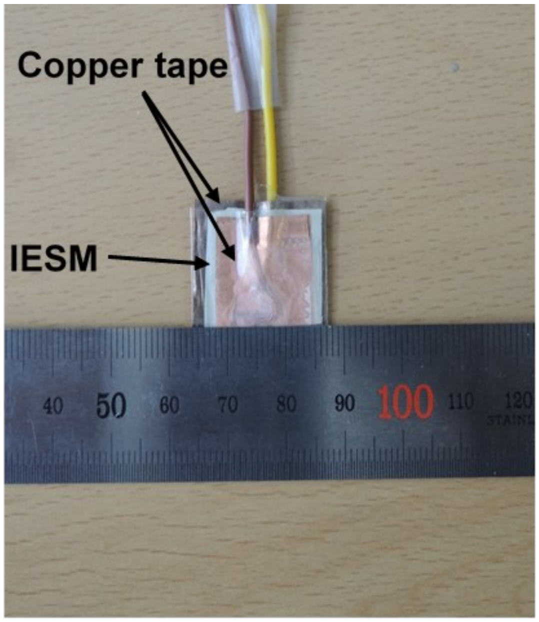

Fig. S2. Photograph of IESM-based piezoelectric function dominant device.

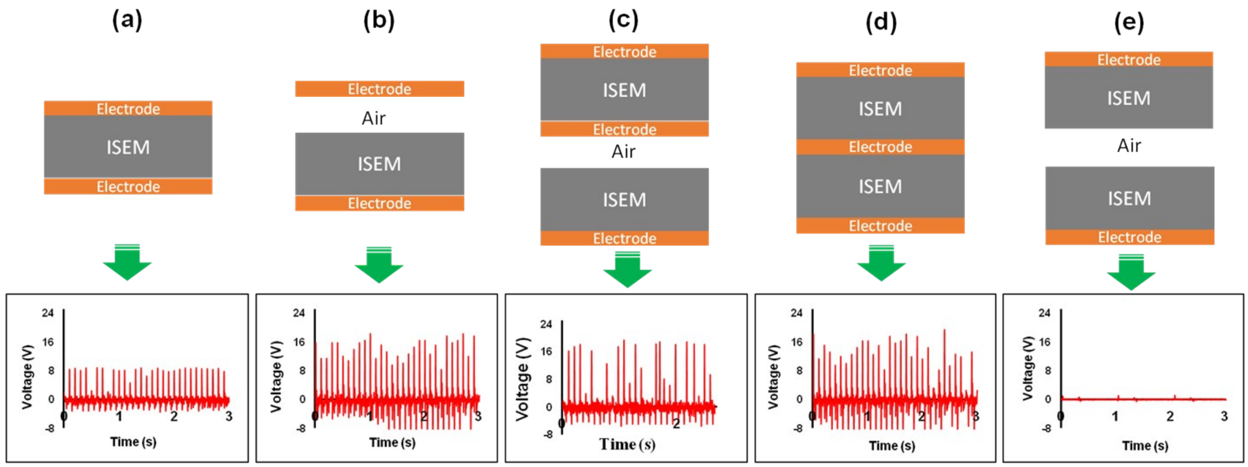

Fig. S3. (a) Piezoelectric without air gap. (b) Triboelectric function dominant device with air gap based study of IESM based self-powered pressure sensor. (c) Hybrid with and without air gap. (d) Two piezoelectric pressure sensors coupled back to back. (e) Air gap between sensing layer.

3. Triboelectric function dominant properties of IESM

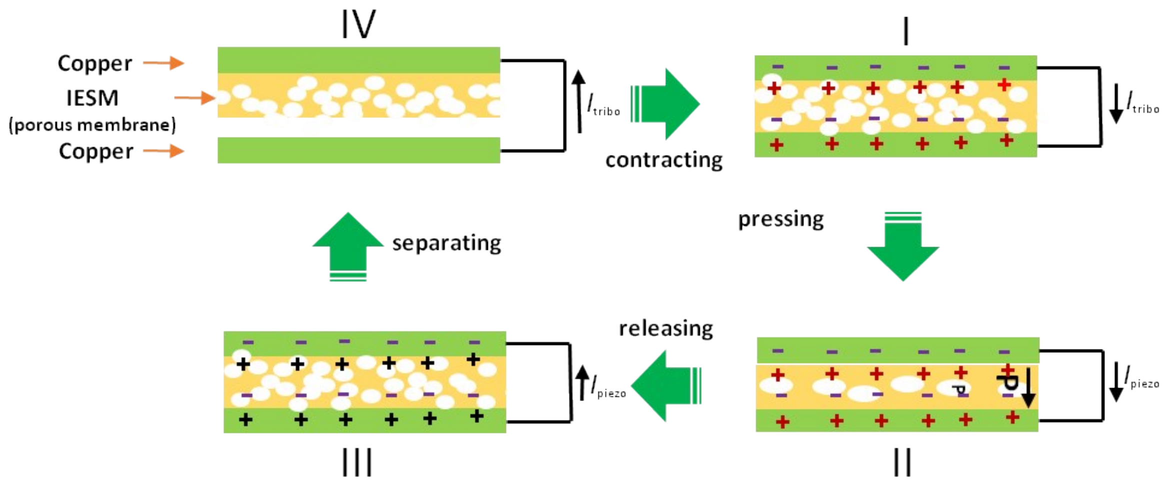

Fig. S4. Stepwise mechanism showing the combined effect for tribo and piezoelectricity. It shows the triboelectric function dominant IESM based sensing device.

When an external pressure is applied across the device, the transfer of charge will take place, as shown in step I. As a result, the triboelectric current will flow across the external circuitry. Upon further increasing the external force, a dipole formation will take place in porous IESM, thus

resulting an additional piezoelectric current flow as discussed in the step II. The device works as a triboelectric function dominant device. The total current will the sum of triboelectric and piezoelectric current flow. The step III and IV depicts the piezoelectric and triboelectric current flow in the reverse direction [2].

4. Pressure measuring analysis

The presented model in Fig. S6 and S8 was used to measure the static and dynamic pressures for IESM based capacitive and self-powered piezoelectric function dominant pressure sensors, respectively.

$$V_{out} = \left(\frac{R_c}{R_a + R_c}\right) V_{in} \qquad (1)$$

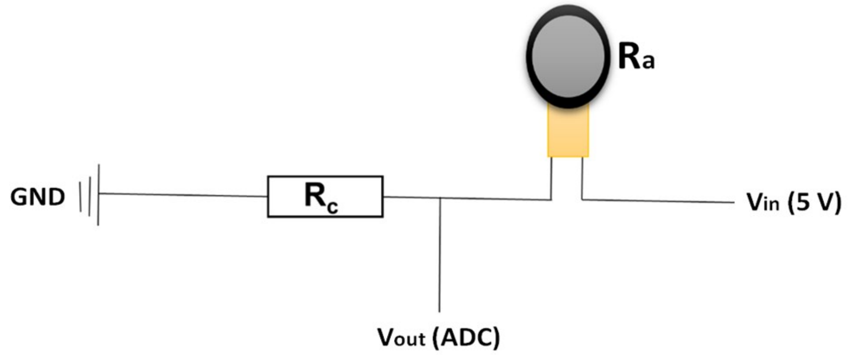

Fig. S5. Homemade pressure measuring unit based on RA9 sensor.

Here, R_c is resistor with the resistance of 2.2 kΩ and R_a is the resistance of the RA9 pressure sensor. The RA9 commercial pressure senor was used to measure the pressure. A homemade pressure measuring unit was used for static pressure measurements as shown in Fig. S5. The calibrated weights were used to define various static pressures applied on the capacitive pressure sensor. The various dynamic pressures were applied on the IESM based pressure sensor controlled by linear stepping motor (Using the stroke test setup based linear servo motor (IRROBOT L12-30PT-4), this experiments are presented in Supplementary movie 3.mp4.). Eq. (1) explains the working phenomenon of the reference sensor. The reference sensor resistance was varied by applied different static and dynamic pressures. At V_{out}, the output voltage also

changed, and the analog output voltage can be converted to digital data using digital to analog conversion (ADC). The gram force can be obtained from a obtained data, the reference sensor has a lower limit of 5 gf (gram force).

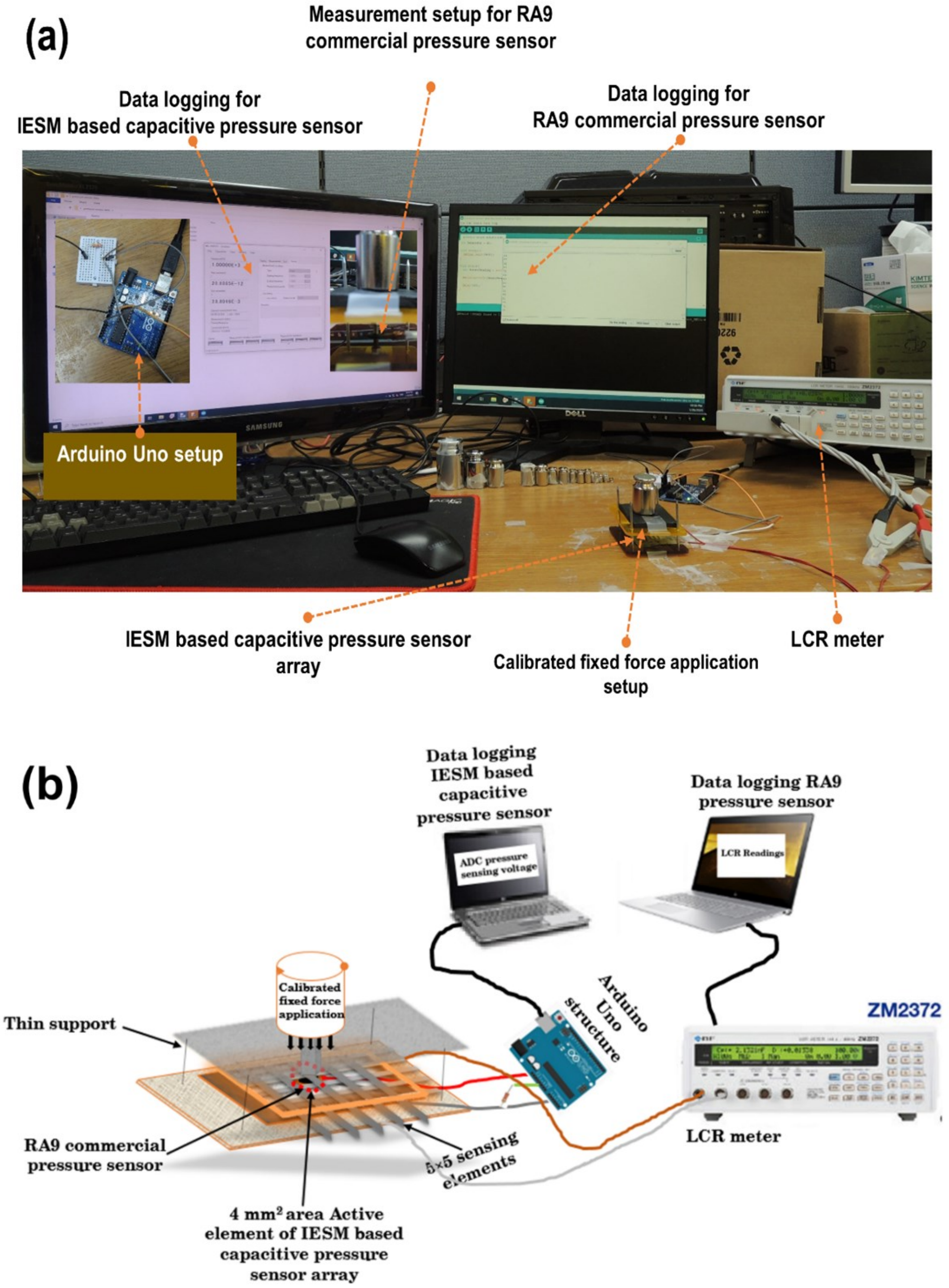

Fig. S6 (a) Pressure sensing setup for IESM based capacitive pressure sensor. (b) The detailed schematics of IESM based capacitive pressure sensor using standard weights.

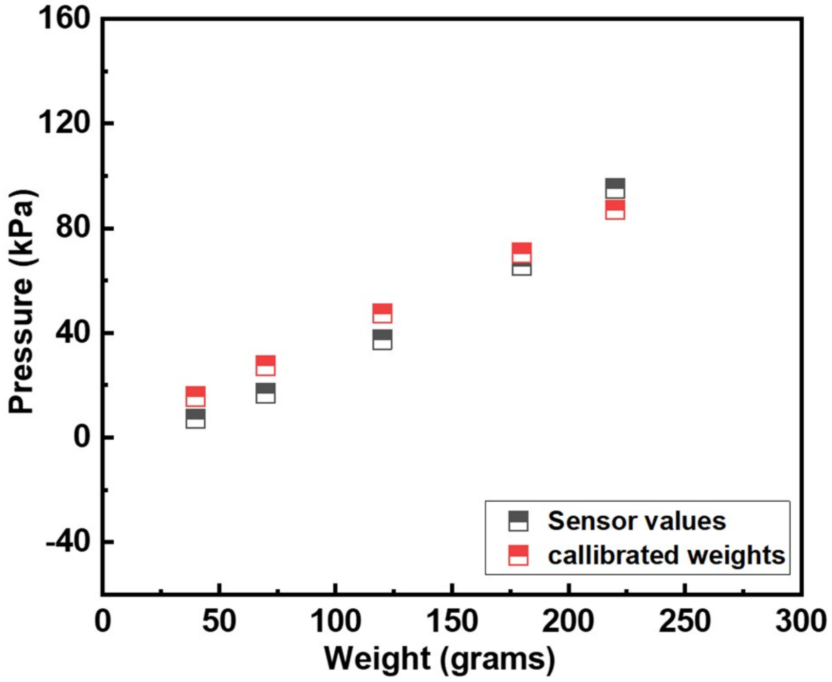

Fig. S7. Calibration test of RA9 commercial pressure sensor.

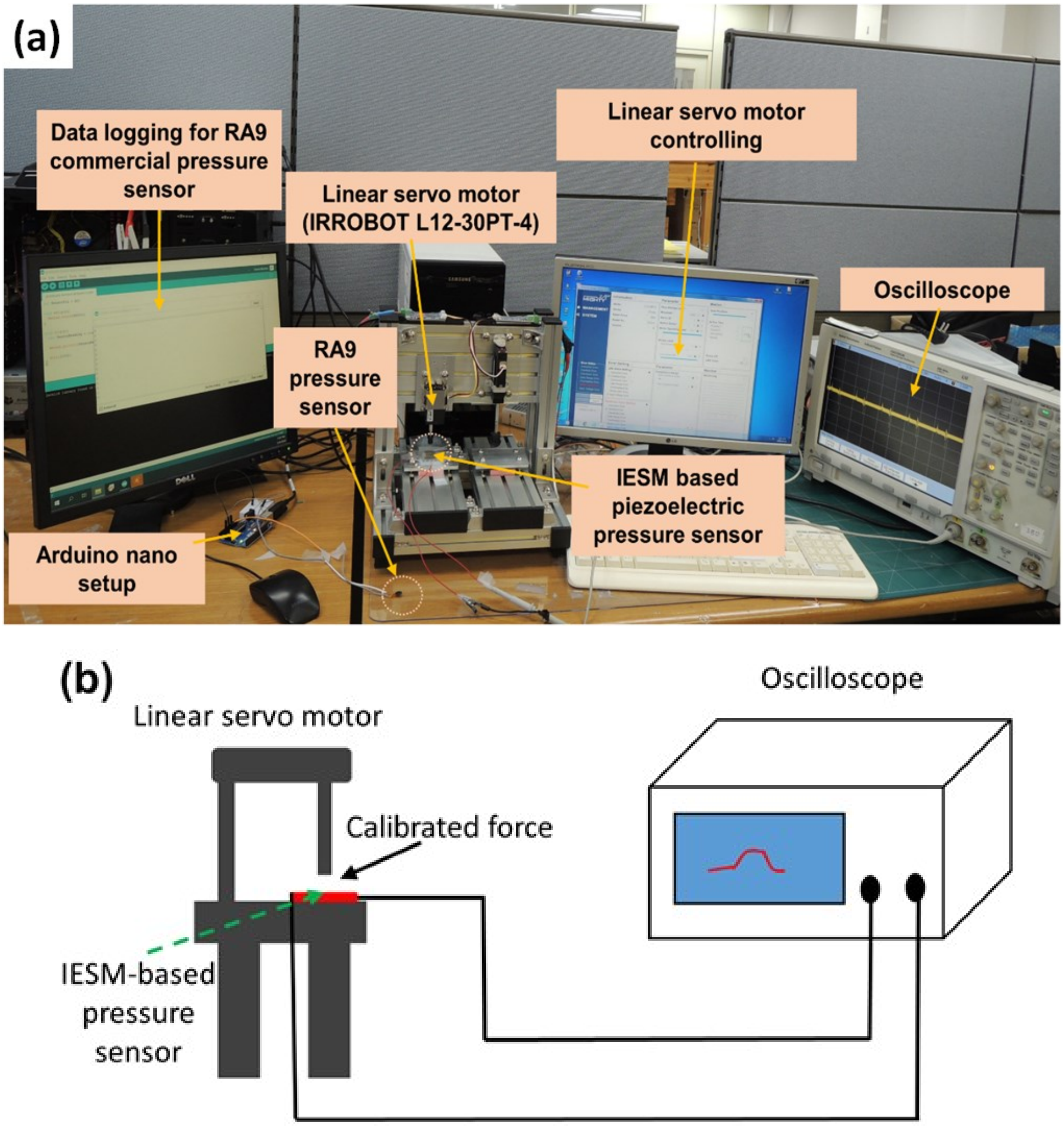

Fig. S8. (a) Pressure sensing setup for IESM based self-powered pressure sensor. (b) Block diagram for testing of IESM based piezoelectric function dominant self-powered pressure sensor. Using the stroke test setup based linear servo motor (IRROBOT L12-30PT-4), this experiments are presented in Supplementary movie 4.mp4.

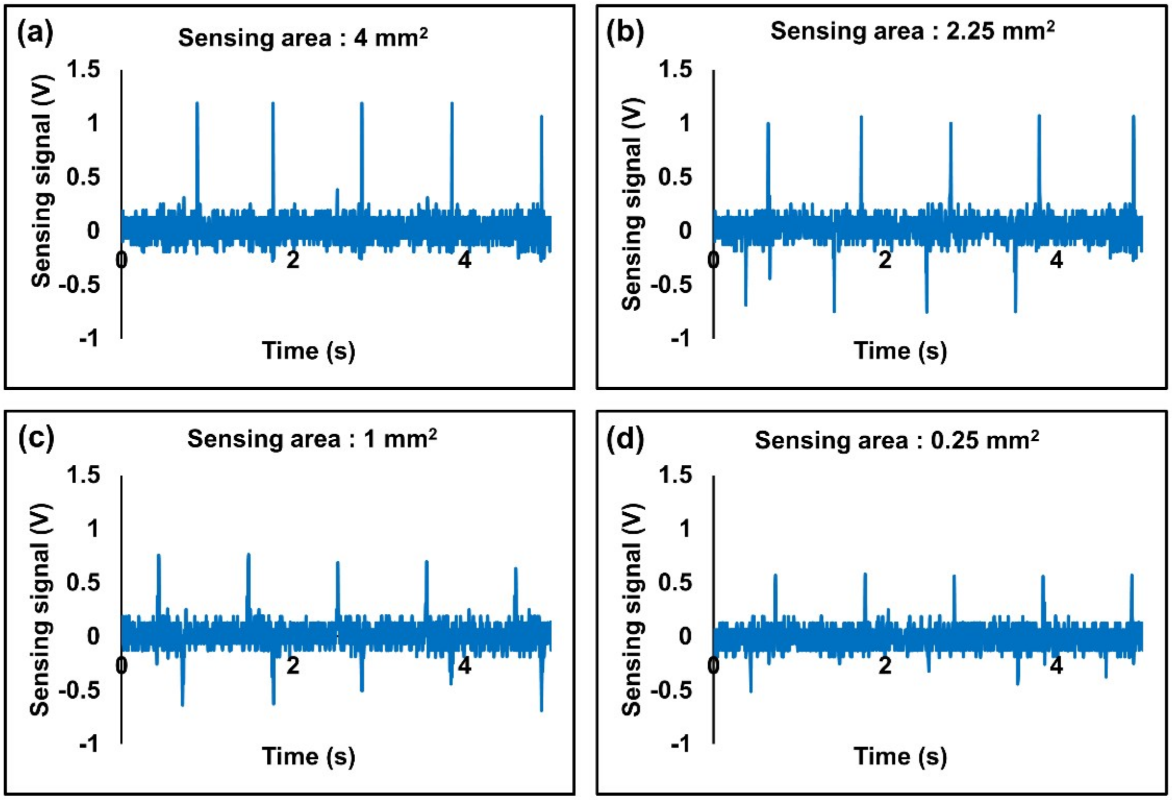

Fig. S9. The attained self-powered voltages against sensing area of 4, 2.25, 1, and 0.25 mm^2, respectively.

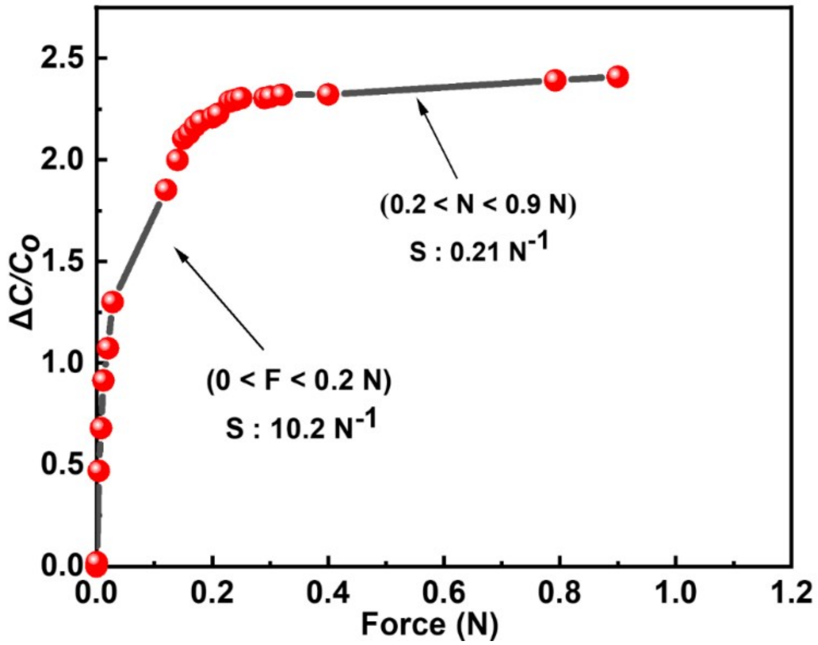

Fig. S10. Relative capacitance change vs Force (N) graph for sensitivity measurements.

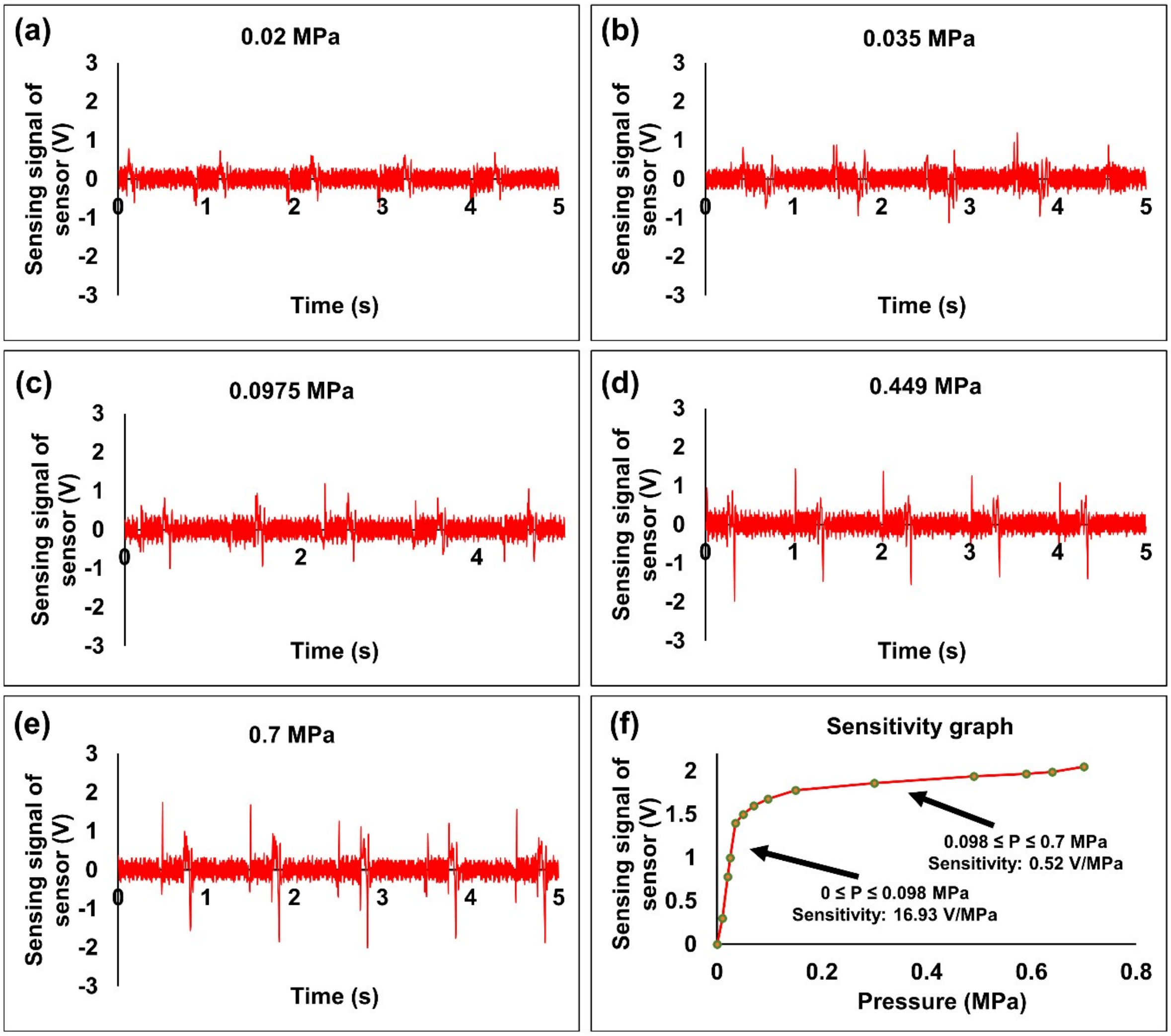

Fig. S11. Sensing signal of the IESM based self-powered piezoelectric function dominant pressure sensor against the applied pressures of (a) 0.02, (b) 0.035, (c) 0.0975, (d) 0.449, and (e) 0.7 MPa. (f) Sensing voltage plotted against various applied pressures.

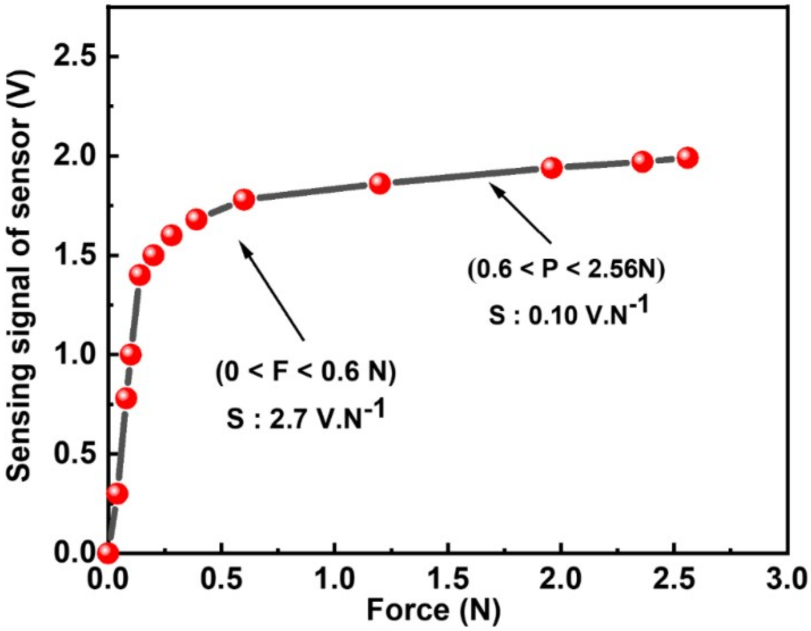

Fig. S12. Sensing signal of sensor vs Force (N) graph for sensitivity measurements.

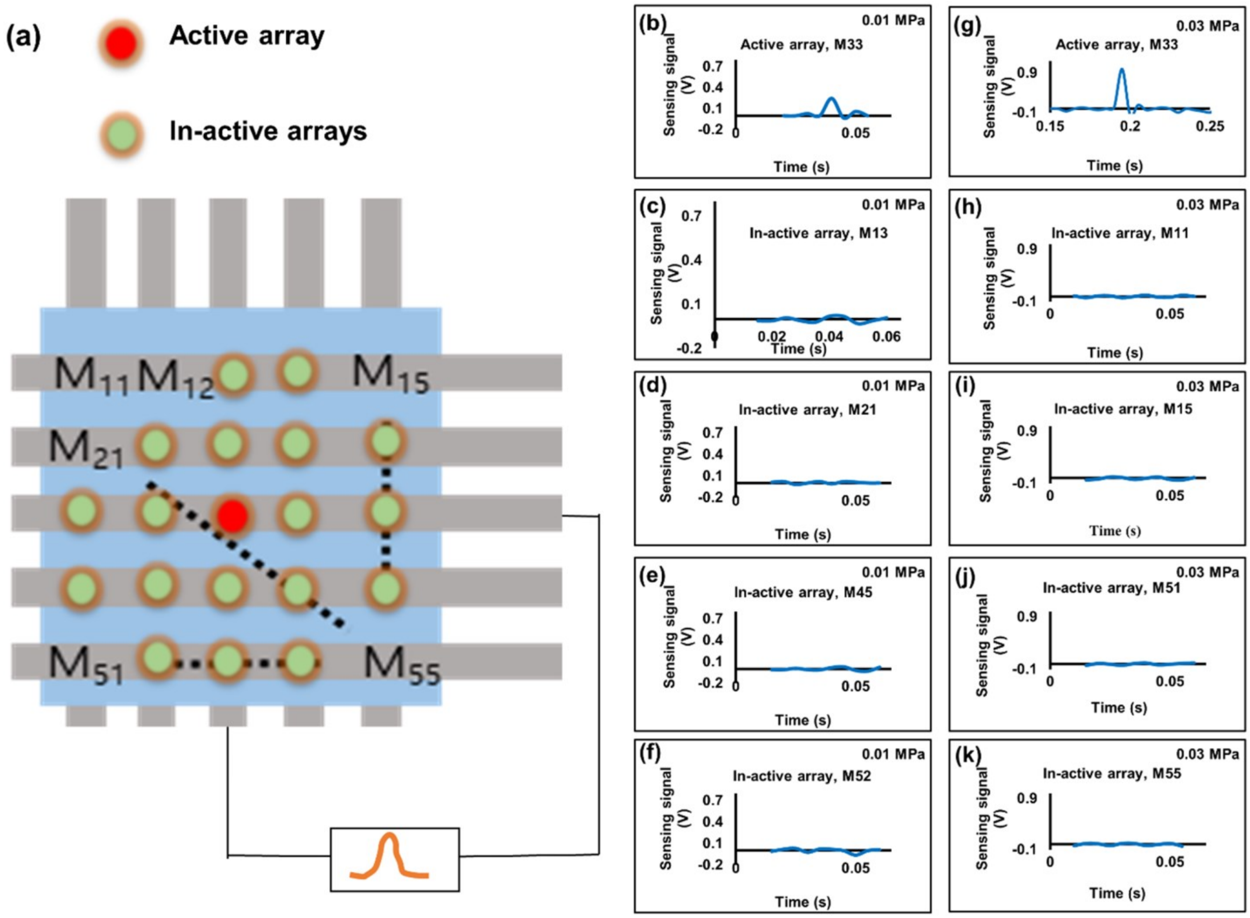

Fig. S13. (a) Sensor array matrix. (b), (c), (d), (e), and (f) are measured signal of M_{33}. M_{13}, M_{21}, M_{45}, and M_{52}, respectively, when 0.01 MPa is stroked across the active and in-active crossbar arrays. (g), (h), (i), (j), and (k) are measured signal of M_{33}. M_{11}, M_{15}, M_{51}, and M_{55}, respectively, when 0.05 MPa is stroked across active and in-active crossbar arrays. Here is shown all sensing elements are independently operated.

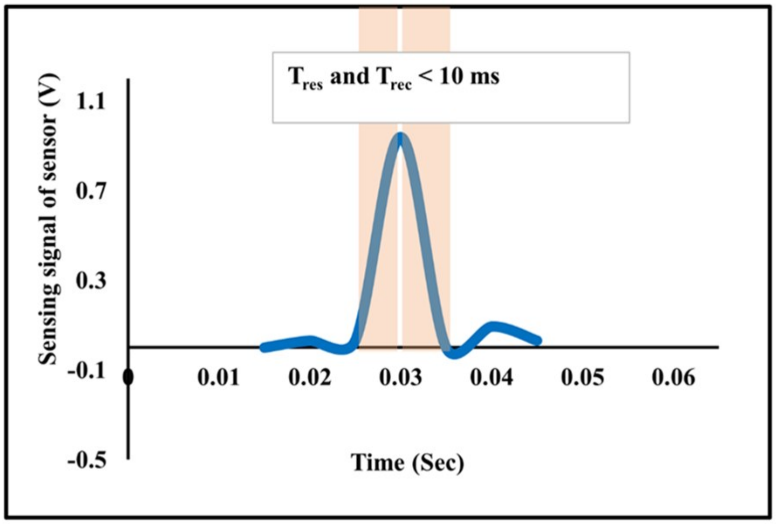

Fig. S14. Response and recovery time of self-powered piezoelectric dominant pressure sensor. The result shows that both the response and recovery time were < 10 ms.

References

[1] M. Han, X. Chen, B. Yu, H. Zhang, Adv. Electron. Mater. 1(10) (2015) 1500187.

[2] G. Suo, Y. Yu, Z. Zhang, S. Wang, P. Zhao, J. Li, X. Wang, ACS Appl. Mater. Interfaces. 8(50) (2016) 34335-34341.
